# Precipitation Drives Frugivory in a Subtropical Generalist Herbivore, the Gopher Tortoise, and Alters Its Functional Role as a Seed Disperser

**DOI:** 10.1002/ece3.70585

**Published:** 2024-11-18

**Authors:** Adrian Figueroa, Pavel Chernyavskiy, Michael Greenacre, Alyssa Herrera, Lydia Cuni, Jennifer Villate, Mauro Galetti, Hong Liu, Steven Whitfield

**Affiliations:** ^1^ Department of Wildlife Ecology and Conservation University of Florida Gainesville Florida USA; ^2^ Department of Earth and Environment Florida International University Miami Florida USA; ^3^ Division of Biostatistics, Department of Public Health Sciences University of Virginia Charlottesville Virginia USA; ^4^ Department of Economics and Business Universitat Pompeu Fabra Barcelona Spain; ^5^ Department of Biological Sciences Florida International University Miami Florida USA; ^6^ Fairchild Tropical Botanic Garden Coral Gables Florida USA; ^7^ Departamento de Biodiversidade, Instituto de Biociências Universidade Estadual Paulista (UNESP) Rio Claro São Paulo Brazil; ^8^ Audubon Nature Institute New Orleans Louisiana USA

**Keywords:** frugivory, gopher tortoise, herbivory, mutualism, plant–animal interactions, seed dispersal

## Abstract

Consumers employ a variety of foraging strategies, and oftentimes the foraging strategy employed is related to resource availability. As consumers acquire resources, they may interact with their resource base in mutualistic or antagonistic ways—falling along a mutualism‐antagonism continuum—with implications for ecological processes such as seed dispersal. However, patterns of resource use vary temporally, and textbook herbivores may switch foraging tactics to become more frugivorous in periods of greater fleshy fruit availability. In this study, we investigated how fleshy fruit consumption of a generalist herbivore—the gopher tortoise ( *Gopherus polyphemus* )—shifts intra‐annually following seasonal precipitation and subsequently examined how this shift toward increased frugivory influences the suite of plant syndromes dispersed. We noted a clear intra‐annual shift toward a more frugivorous diet which coincided with seasonal precipitation and subsequently observed a marked shift in the plant syndromes dispersed with increasing frugivory. We found that as this generalist herbivore became more frugivorous, it dispersed a greater variety of plant syndromes at low levels of frugivory. However, when the gopher tortoise exhibited high levels of frugivory, the seed load was dominated by those exhibiting the endozoochory syndrome. This study illustrates a functional shift in a seed‐dispersing herbivore toward that of a classical frugivore, suggesting that temporal variation in foraging strategy and the temporal scale in which foraging habits and seed dispersal interactions are quantified have implications for the suite of plant syndromes species disperse. Furthermore, trade‐offs may exist that provide plants with the endozoochory syndrome with a competitive advantage over seeds with contrasting traits, such as the foliage is the fruit syndrome which is expected to experience greater dispersal by classical herbivores.

## Graphical abstract

A generalist herbivore, the gopher tortoise, becomes more frugivorous intra‐annually, following seasonal precipitation. As it increasingly becomes frugivorous, it disperses seeds exhibiting very different dispersal syndromes. At high levels of frugivory, this species behaves as a classical frugivore with regard to the types of seeds it disperses. It accomplishes this by dispersing more seeds of fleshy‐fruited plants and fewer seeds of plants with the Foliage is the Fruit dispersal syndrome, exhibiting a shift in its functional role as a seed disperser.

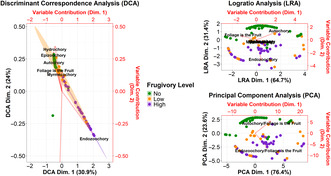

## 1 Introduction

Resource utilization is a fundamental ecological process that mediates a variety of interactions, from mutualisms to antagonisms (Bronstein 2015). Investigating resource use patterns provides insight into the trophic niche species occupy and the functional roles they may play in an ecosystem (Elton 2001; Chase and Leibold 2009). Furthermore, quantifying spatiotemporal patterns of resource use can reveal shifts in the dietary habits of consumers toward preferred food items that are limited in time and/or space (Abrahms et al. 2021). One spatiotemporally limited resource that is closely tracked by animals is fleshy fruits (Koike et al. 2008; Takahashi et al. 2008). While botanically, the consumption of any fruit type is considered frugivory, we hereafter refer to frugivory in an ecological sense, specifically as the consumption and passage of seeds, pulp, and skin from fleshy fruits (Howe and Smallwood 1982; Van der Pijl 1982; Howe 1986; Jordano 2000). In many ecosystems, as fleshy fruits become more abundant through time, animals shift their diet to become more frugivorous (Remis 1997; Herrera et al. 2008; Robira et al. 2023).

Consequently, as consumers become more frugivorous by increasingly ingesting fleshy fruits, they may also become more effective seed dispersers for these species by more frequently dispersing their seeds (Schupp 1993; Schupp, Jordano, and Gómez 2010; Marques Dracxler and Kissling 2022), so long as they do not predate on the seeds themselves. Since seed dispersal is a fundamental aspect of the life cycle of plants (Traveset, Heleno, and Nogales 2014), quantifying the prevalence of fleshy fruits in the diets of consumers is a first step toward understanding their functional roles as seed dispersers and where they fall along the mutualism‐antagonism continuum (see van Leeuwen et al. 2022).

Although plant dispersal syndromes alone have shown to be unreliable in predicting the ingestion and dispersal (i.e., endozoochorous dispersal) of seeds (Green, Baltzinger, and Lovas‐Kiss 2021), studies that determine how the functional role of seed dispersers may change with temporal dietary shifts could reveal the interplay between foraging strategies and the ecological role of consumers as seed dispersers. In systems where the phenology of fleshy fruit‐bearing plants is linked to seasonal phenomena like precipitation (Bancroft, Bowman, and Sawicki 2000; Redwine et al. 2007), one approach could be to quantify temporal changes in these factors along with fleshy fruit consumption by the seed disperser of interest.

Subsequently, one could test if frugivory in the seed disperser corresponds with seasonal phenomena and consequently if the degree of frugivory influences the number of seeds dispersed from plants of different syndromes (Howe and Smallwood 1982; Van der Pijl 1982). Although consumers may become more frugivorous seasonally, it does not necessarily mean that the number of seeds of other syndromes dispersed should change. That is, unless there is indeed a competitive advantage to plants exhibiting the endozoochory (i.e., fleshy fruit) syndrome.

In this study, we investigate patterns of frugivory in a population of herbivorous hindgut fermenters, the gopher tortoise ( *Gopherus polyphemus* ), and aim to address whether its degree of frugivory is linked to seasonal patterns of precipitation and whether its functional role as a seed disperser changes as it becomes more frugivorous. Specifically, we aim to address the following questions:

1. Is there a temporal pattern of frugivory in this species, and if so, is it related to seasonal precipitation?
2. If shifts toward frugivory are associated with precipitation, what is the time lag between precipitation and frugivory?
3. As frugivory increases, does the suite of plant syndromes dispersed change?
4. What dispersal syndromes are most affected by functional shifts in seed dispersal with increasing frugivory?

Considering the phenology of many fleshy fruit‐producing plants in south Florida coinciding with seasonal rains (Redwine et al. 2007; Lodge 2017), as well as the 2‐ to 3‐week gut retention time of the gopher tortoise (Bjorndal 1987), we hypothesize that there will be a time lag between seasonal precipitation in south Florida and frugivory in the gopher tortoise on the order of months. This time lag would allow for the plant community to produce fleshy fruits and for the gopher tortoises to find, ingest, and egest them. We expect that as the gopher tortoise becomes more frugivorous, it will increasingly disperse more seeds of plants with the endozoochory dispersal syndrome but will continue to disperse similar numbers of seeds of other plant syndromes given its extremely broad diet (Birkhead et al. 2005; Ashton and Ashton 2008; Moore and Dornburg 2014).

## 2 Materials and Methods

### 2.1 Site Description

Our study was conducted in Miami, Florida, USA, in the globally imperiled pine rockland ecosystem that surrounds Zoo Miami at The Richmond Tract (USFWS 1999; Possley et al. 2018, 2020). The Richmond Tract is a complex of properties that spans 830‐ha and contains the largest extent of pine rockland habitat outside of Everglades National Park (Bradley and Gann 2005). The pine rockland is the most biodiverse ecosystem in South Florida with over 430 native plant species and a multitude of large vertebrates that have largely been extirpated as a result of defaunation and urban development (Dirzo et al. 2014; Lodge 2017; Trotta et al. 2018; Figueroa et al. 2023). This ecosystem is fire‐maintained and characterized by a sparse, savanna‐like canopy of endemic south Florida slash pine ( *Pinus elliottii var. densa* ) with rare and endemic herbs, as well as grasses, euphorbs, and succulents interspersed between an understory of shrubs and palms (Possley, Woodmansee, and Maschinski 2008; Diamond and Heinen 2016).

South Florida is the ideal setting for this study due to its oscillation between wet and dry seasons, which triggers seasonal fires in the dry‐to‐wet season transition (Slocum et al. 2010; Platt, Orzell, and Slocum 2015). During this transitory period and well into the wet season, many plants across South Florida flower and set fruit, particularly in species that produce fleshy fruits (i.e., exhibiting the endozoochory dispersal syndrome) (Bancroft, Bowman, and Sawicki 2000; Redwine et al. 2007). In addition to the diversity of plants they contain, pine rocklands provide habitat for several states and federally listed fauna (USFWS 1999). One of these animals is the gopher tortoise—a longtime inhabitant of the pine rockland ecosystem that persists in remnant preserves to this day (Simpson 1920; Carr 1940; Monroe 1943; Enge, Robson, and Krysko 2004; Whitfield et al. 2018, 2022; Figueroa, Lange, and Whitfield 2021).

### 2.2 Study Species

The gopher tortoise is the only native tortoise found east of the Mississippi River (Auffenberg and Franz 1982; Bury and Germano 1994; Edwards et al. 2016). Its range spans the southeastern United States, from Louisiana to South Carolina and south into Miami‐Dade County and Cape Sable in Florida (Kushlan and Mazzotti 1984; Enge, Robson, and Krysko 2004; Waddle, Mazzotti, and Rice 2006; Whitfield et al. 2024). Gopher tortoises support over 350 commensal animal species that use their burrows (Diemer 1986; Lips 1991) and are known to forage on over 1000 plant species across their range (Ashton and Ashton 2008).

Many studies have investigated the diet and foraging ecology of this species (McRae, Landers, and Garner 1981; MacDonald and Mushinsky 1988; Mushinsky, Stilson, and McCoy 2003; Ashton and Ashton 2008), classifying it as an herbivore that opportunistically engages in frugivory (Birkhead et al. 2005; Hanish 2018; Richardson and Stiling 2019a, 2019b). As such, it is a widely‐recognized seed disperser by dispersing the seeds of fleshy‐fruited (Hanish 2018; Richardson and Stiling 2019a), as well as species that exhibit the “Foliage is the Fruit” dispersal syndrome (*sensu* Janzen 1984, Carlson, Menges, and Marks 2003, Birkhead et al. 2005, Figueroa, Lange, and Whitfield 2021), oftentimes enhancing seed germination (Falcón, Moll, and Hansen 2020). Furthermore, gopher tortoises can have home ranges spanning over 1 ha and are known to forage up to 40 m away from their burrows (McRae, Landers, and Garner 1981; Eubanks, Michener, and Guyer 2003), potentially dispersing seeds far from their parent plants and allowing them to escape density dependent. Additionally, male gopher tortoises can travel distances upwards of 500 m in search of females (Guyer, Johnson, and Hermann 2012), potentially providing long‐distance dispersal services for the plants whose seeds they consume (Nathan and Muller‐Landau 2000).

**FIGURE 1:**
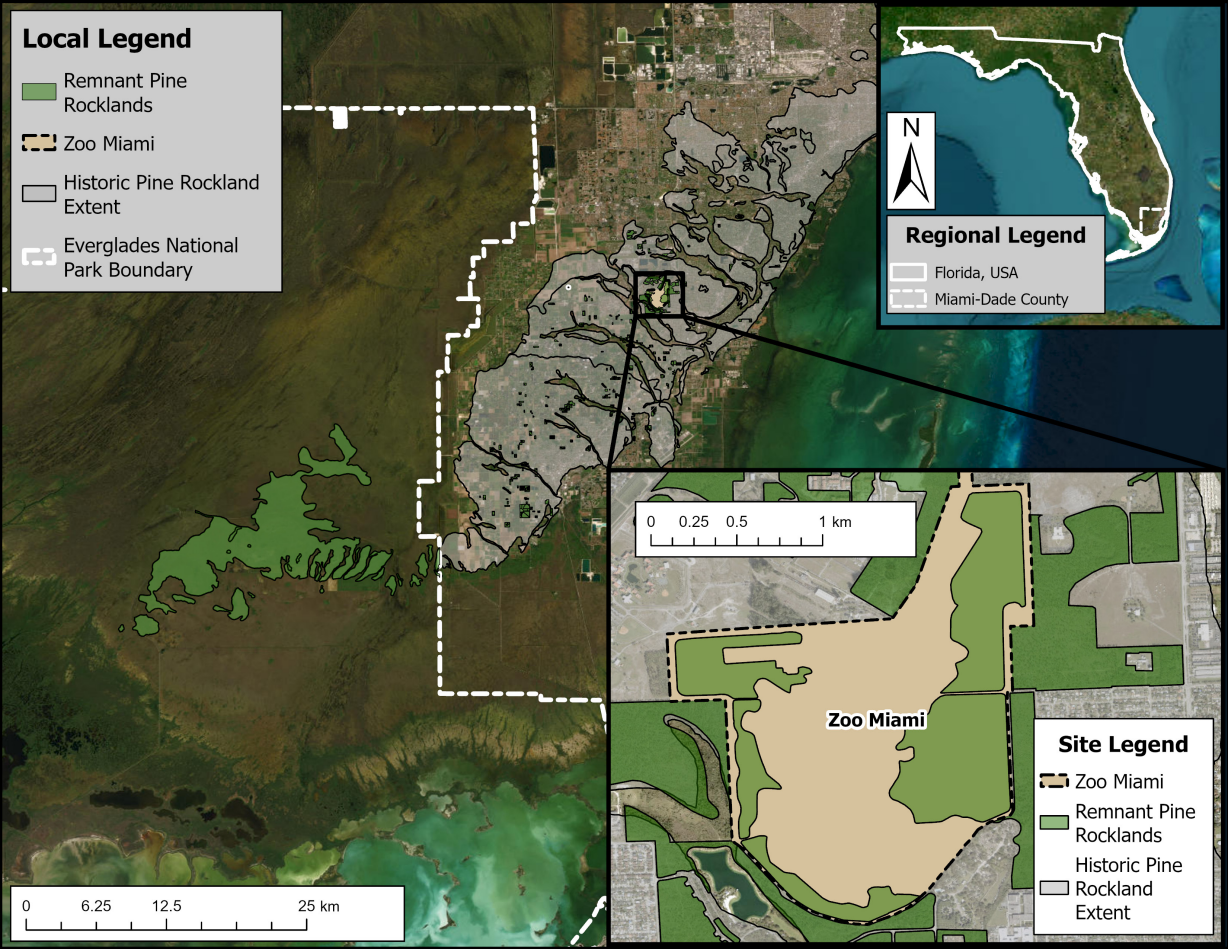
Our study site, Zoo Miami, lies on the largest expanse of pine rockland habitat outside of Everglades National Park known as The Richmond Tract in Miami‐Dade County, Florida, USA.

The gopher tortoise thus serves as a model species for investigating how frugivory might fluctuate temporally in a generalist seed‐dispersing herbivore, providing an opportunity to quantify how its frugivory varies temporally and whether its functional role as a seed disperser changes. The tortoises in this study (*n* = 21) are individually marked wild individuals found in three aggregations which we refer to as the East, South, and West sites—named after the cardinal directions in which they are located across the pine rockland habitat surrounding Zoo Miami (see Figure 1). These tortoise aggregations are due to a combination of the species' social behavior (Guyer, Johnson, and Hermann 2012), as well as the geology of this ecosystem (Hoffmeister, Stockman, and Multer 1967), which can limit the availability of deep sandy soils that facilitate burrowing (Whitfield et al. 2022). During the study, no tortoises migrated from one site to another, as we regularly tracked individuals via radio telemetry, so each site has a perfectly nested subset of individuals that occupy it. While formal surveys were not conducted, the plant communities in both the South and West sites were representative of managed pine rockland habitat while the East site had a greater presence of invasive plant species such as Burma reed ( *Neyraudia reynaudiana* ), showy rattlebox ( *Crotalaria spectabilis* ), and shrub verbena ( *Lantana camara* ).

### 2.3 Study Design

#### 2.3.1 Scat Collection and Dissection

We conducted this study over a 1.5‐year period with scat collection beginning on May 11, 2021, and ending on November 9, 2022. We tracked wild gopher tortoises at the Richmond Tract twice weekly via radio telemetry. If an individual defecated during handling, we collected the samples and labeled it with the tortoise's ID number, location, and the date of collection. Sometimes, we encountered fresh fecal samples belonging to unknown tortoises and thus collected them. For these samples, we marked the GPS location, noted the date, and labeled each with a unique identifier indicating it came from an unknown individual.

Fecal samples were either dissected on the same day of collection or frozen for dissection at a future date. We performed scat dissections with forceps over laminated graph paper (29.59 × 21.01 cm) containing 5 × 5 mm grids as a static background reference to compare the relative contributions of food items to the total fecal volume. All food items recovered from fecal samples were identified to the lowest taxonomic unit or qualified as their own category (e.g., fur/hair was recovered from multiple species and subsequently categorized as mammal fur). We then aggregated the food items into five functional food categories—grasses, legumes, other plants, fleshy fruit, and animal remains based on their taxonomic identity and importance in the gopher tortoise diet (Birkhead et al. 2005; Ashton and Ashton 2008; Moore and Dornburg 2014; Hanish 2018).

We then visually estimated the relative contribution of each food category to the total scat volume; this approach has been compared with other methods resulting in its acceptance for dietary studies (Klare, Kamler, and MacDonald 2011). We recorded the contribution of each food category to the total fecal volume as proportions of either 0.01, 0.05, or in increments of 0.05 all the way to the total scat volume of 1.00. If a value less than 0.05 remained after quantifying the relative contribution of all food items, we would allocate the amount to the most abundant food category in the sample.

While other studies have used activity budgets to quantify the strength of frugivory (Pavelka and Knopff 2004), we directly measured frugivory as the proportion of total fecal volume comprised of fleshy fruit in the fleshy fruit food category. As a result, we focus our analyses on the fleshy fruit category exclusively, and hereafter refer to it as the “degree of frugivory,” as this quantifies fleshy fruit consumption.

#### 2.3.2 Classification of Dispersal Syndrome

To ensure thorough extraction of seeds, we carefully combed through fecal contents using forceps and an illuminated AmScope SM‐2 series trinocular stereo microscope (7×–45× magnification). Mounted to the microscope was a 14‐megapixel AmScope MU1403 high‐performance digital camera, which facilitated the viewing, counting, and photographing of seeds needing further identification. Seeds were identified to the lowest taxonomic unit using dichotomous keys, online references, and consultations with local botanists (Gann et al. 2001; Gann, Bradley, and Woodmansee 2002; Wunderlin et al. 2016; Flora of North America Editorial Committee, eds. 1993+ 2023). All seeds were counted and those identified to the species level had their dispersal syndromes recorded according to criteria from Howe and Smallwood (1982), Van der Pijl (1982), and Janzen (1984) (see Table 1). In samples containing exceptionally high numbers of small seeds (e.g., *Buchnera americana* , *Euphorbia hirta* , *Mosiera longipes* ), where it was impractical to manually count every seed, we aggregated all seeds within the frame of view of the microscope and extrapolated to the rest of the sample (see Data S1 for further details). To ensure only potentially viable seeds were considered, we recorded whether the seeds appeared to be intact or were obviously scarified/damaged. Only seeds in the intact category were used in our analyses.

**TABLE 1:** Criteria for classifying the plant dispersal syndromes using guidance from Howe and Smallwood (1982), van der Pijl (1982), and Janzen (1984).

Criteria for classification of plant dispersal syndromes			
Syndrome	Adapted mechanism of dispersal	Indicative structures (on fruit or seeds)	References
Anemochory	Wind‐dispersal	Plumes or wings	Howe and Smallwood (1982), van der Pijl (1982)
Autochory	Self‐dispersal	Dehiscing or exploding fruits/seed pods	van der Pijl (1982)
Endozoochory	Ingestion by animals	Fleshy structures in the form of an aril, pericarp, or pulp.	Howe and Smallwood (1982), van der Pijl (1982)
Epizoochory	Adhesion to animal hairs/feathers	Hooks, barbs, or other clingy appendages	Howe and Smallwood (1982), van der Pijl (1982)
Foliage is the Fruit	Ingestion by herbivores	Seeds enveloped in nutritive leaves/foliage	Janzen (1984)
Hydrochory	Water‐dispersal	Small, light seeds capable of floatation and/or unwettable	Howe and Smallwood (1982), van der Pijl (1982)
Myrmecochory	Ant‐dispersal	Fatty appendages known as elaiosomes	Howe and Smallwood (1982), van der Pijl (1982)
Synzoochory	Scatter‐hoarding	Cacheable fruits/nuts typically in the Fagaceae	van der Pijl (1982)

#### 2.3.3 Precipitation Data Collection

To collect data on seasonal precipitation, we accessed the online Florida Climate Center database from Florida State University and downloaded daily precipitation data from a nearby National Oceanic and Atmospheric Administration cooperative meteorological station (25.5819, −80.4361), located less than 5 km from our study site. To ensure the data were relevant to our study, we selected data in a search window spanning from May 1, 2021 to November 30, 2022, encompassing all precipitation during our study period. The daily precipitation for each month was summed to obtain total monthly precipitation values. For months with data collected in both 2021 and 2022, we calculated the mean of the total monthly precipitation.

### 2.4 Statistical Analysis

All statistical analyses were carried out in R version 4.3.2 using various packages explicitly stated in the following subsections (R Core Team 2022).

#### 2.4.1 Seasonal Patterns of Precipitation and Frugivory

For the research question on the relationship between time and precipitation, we constructed a generalized additive model (GAM) to investigate total monthly precipitation as a nonlinear function of the calendar month (Pedersen et al. 2019). The GAM was estimated using the Bayesian brms package (Bürkner 2017, 2018), specifying a normal distribution for the response variable (precipitation in cm) with the default uninformative priors from the brms package. We included flat priors for regression coefficients (with vectorization for specific months), and Student's *t*‐distributions with 3 degrees of freedom for the intercept, standard deviations, and sigma, where the intercept has a mean of 13.3 and a scale of 11.4, while the standard deviations and sigma have a mean of 0 and a scale of 11.4.

Similarly, we modeled the relationship between time and frugivory through a GAM where the calendar month was the predictor and the degree of frugivory (a continuous proportion) was the response variable. For this analysis, we utilized the Bayesian ordbetareg package which models continuous proportion variables, while allowing for possible values of exactly 0 and/or exactly 1 (Kubinec 2022). In this model, we also specified the default uninformative priors. These uninformative priors included normal priors with a mean of 0 and standard deviation of 5 for the regression coefficients (vectorized for specific months), induced Dirichlet distributions for categorical cuts, a Student's *t*‐distribution with 3 degrees of freedom for the intercept and standard deviations (mean 0, scale 2.5), and an exponential distribution with a rate of 0.1 for the parameter phi.

#### 2.4.2 Time Lag Between Precipitation and Frugivory

Since frugivory can only occur after fleshy fruits become available, there is a natural time lag between precipitation and fruit appearance in fecal samples. To explore this time lag, we performed a cross‐correlation analysis using the astsa package (Shumway, Stoffer, and Stoffer 2000). The cross‐correlation function (CCF) was computed to examine the association between average monthly precipitation and the mean degree of frugivory in each month across lags from −12 to +12 months. This range accounts for the variable time it takes the plant community to flower and produce fruits (van Schaik, Terborgh, and Wright 1993), and the gopher tortoise's gut passage rate, which is typically around 2–3 weeks (Bjorndal 1987).

To formally test the relationship between lagged precipitation and frugivory, we estimated a GLM through the ordbetareg package with the default uninformative priors and the ordered beta distribution family. The priors included a normal distribution with a mean of 0 and standard deviation of 5 for regression coefficients, vectorization for lagged average monthly precipitation, Dirichlet distributions for categorical cuts, a Student's *t*‐distribution with 3 degrees of freedom for the intercept (mean 0, scale 2.5), and an exponential distribution with a rate of 0.1 for the parameter phi. We specified monthly precipitation as the predictor variable with a time lag based on the results of the CCF, and we specified the degree of frugivory as the response variable. After running the model, we examined the model diagnostics and summary statistics using Bayesian measures of effect size and existence implemented in bayestestR (Makowski, Ben‐Shachar, and Lüdecke 2019; Makowski et al. 2019).

#### 2.4.3 Functional Shift in Seed Dispersal With Increasing Frugivory

To investigate whether frugivory influences the gopher tortoise's functional role as a seed disperser, we first categorized the levels of frugivory by calculating all quartiles for the degree of frugivory. The first and second quartiles had a value of 0.00, which combined to become the “No” frugivory category. The third quartile had a value of 0.05, so values > 0.00 and ≤ 0.05 became the “Low” frugivory category, and the fourth quartile had a value of 0.99, so values that were > 0.05 and ≤ 1.00 became the “High” frugivory category. In addition to the quartile values, considering that the gopher tortoise is a primarily herbivorous species (Ashton and Ashton 2008), we determined it was adequate for samples with > 0.05 of fecal volume comprised of fleshy fruit to be considered “High” frugivory.

We then aggregated seed counts for all species exhibiting the same dispersal syndrome. This process provided us with total seed counts for each of the syndromes listed in Table 1 within each fecal sample. We then normalized the seed counts for each syndrome as the proportion of all seeds dispersed in each sample using the formula $\frac{x_{i}}{\sum_{i=1}^{n}x_{i}}$, where 𝑥_i_ is the number of seeds of dispersal syndrome *i* in the sample of interest, for all *n* dispersal syndromes. This transformation resulted in a compositional dataset, ideal for performing correspondence analysis (CA), which we conducted using the easyCODA package (Greenacre 2019).

CA is a multivariate technique that ordinates compositional data, allowing for the visualization of the associations between grouping variables and the various parts of the composition (Greenacre 2017). In our case, the grouping variable is the frugivory level. We ordinated the normalized seed counts in the CA, plotted the 99% confidence intervals for each frugivory level (No, Low, and High), and followed the ordination with a permutational multivariate analysis of variance (PERMANOVA) using the vegan package (Oksanen et al. 2022). This was done to test for differences in the seed dispersal syndromes dispersed based on the level of frugivory exhibited. The PERMANOVA was performed on the original count data, given its suitability for analyzing ecological count data.

We performed the PERMANOVA with 10,000 permutations based on Bray–Curtis dissimilarity. Additionally, we created a distance matrix using the Bray–Curtis method and performed a multivariate homogeneity of group dispersions analysis (PERMDISP) to assess dispersion differences between frugivory levels. An ANOVA was conducted on the PERMDISP object, followed by a Tukey post hoc test to determine which frugivory levels differed significantly in the seed syndromes dispersed.

## 3 Results

### 3.1 Seasonal Patterns of Precipitation and Frugivory

**FIGURE 2:**
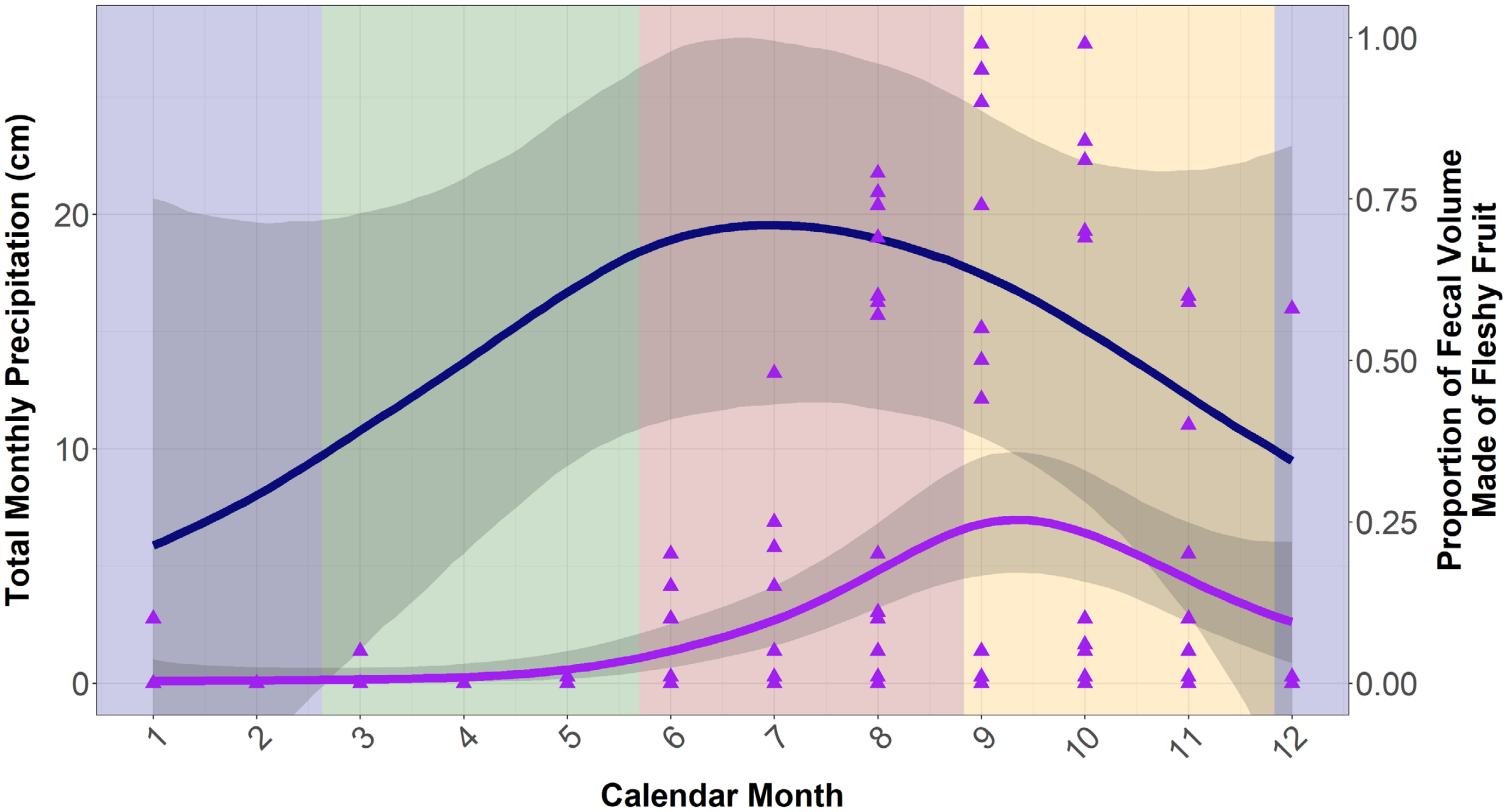
Seasonal patterns of precipitation (blue line) and frugivory (purple line) with 95% credible intervals in gray. Purple triangles represent each fecal sample and the colored rectangles in the background represent the calendar seasons. Winter is blue, spring is green, summer is red, and fall is orange. The horizontal axis is the calendar month of the year, the left vertical axis is the total monthly precipitation for a given month, and the right vertical axis is the degree of frugivory.

In total, we collected 180 fecal samples from 24 known individuals and 27 samples from unknown tortoises for a total of 207 samples. Of the 207 fecal samples, 72 of them (34.78%) exhibited frugivory in at least trace amounts while the remaining 135 (65.22%) showed no signs of frugivory. The GAM revealed notable increases in monthly precipitation throughout the spring and summer months with a clear peak of close to 20 cm of rainfall in July (Figure 2). Subsequently, we observed an increase in frugivory through the late spring and entire summer before peaking in the early fall (Figure 2). At its peak in September, fleshy fruits comprised approximately a quarter of the gopher tortoise diet (24% of fecal volume) and fruit consumption persisted throughout much of the fall before declining to nearly nonexistent levels in the winter.

### 3.2 Time Lag Between Precipitation and Frugivory

**FIGURE 3:**
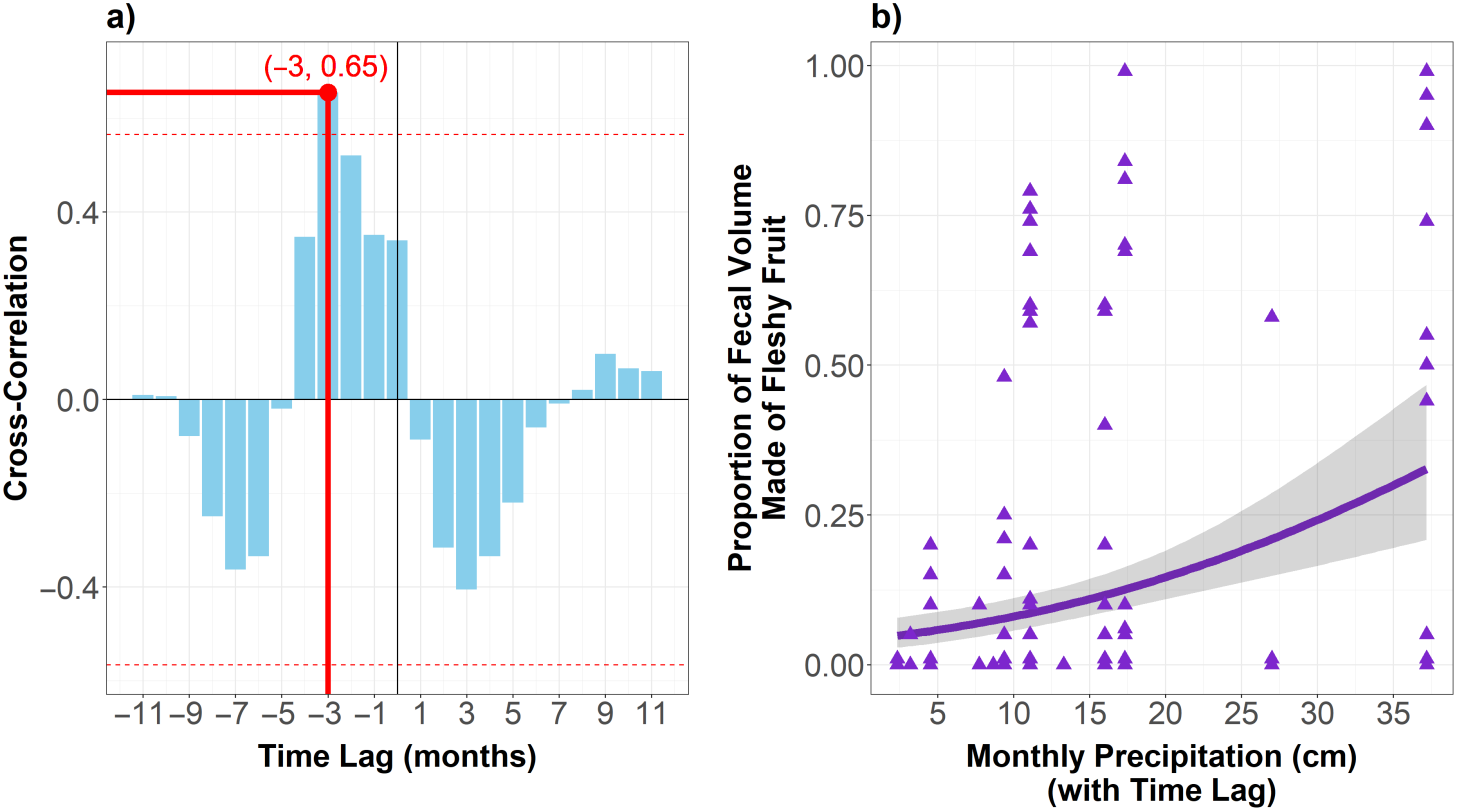
(a) Cross‐correlation analysis illustrating the monthly lags backward (indicated by negative values) and forward (indicated by positive values) in time between total monthly precipitation and the mean frugivory exhibited in that month. The dashed red lines depict the 95% confidence bands around a cross‐correlation of 0.00, with bars exceeding the range being statistically significant. The solid red lines pinpoint the value with the highest correlation coefficient, being the −3 lag with a correlation coefficient of 0.65. (b) Generalized linear model (GLM) regression model depicting the relationship between 3‐month‐lagged average monthly precipitation on the horizontal axis and the degree of frugivory on the vertical axis.

The cross‐correlation analysis revealed the highest correlation at a lag of −3 months, with a significant correlation coefficient of 0.65 (Figure 3a). This suggests that frugivory was most strongly correlated with precipitation occurring 3 months prior, meaning that after seasonal rains begin it takes about 3 months—or a full season—for fleshy fruits to subsequently appear in the fecal contents of the gopher tortoise. The GLM relating lagged precipitation to frugivory demonstrates that monthly precipitation with a 3‐month time lag is a strong predictor of frugivory (Figure 3b). The median effect of lagged monthly precipitation on frugivory is 0.04, with a credible interval (CI) from 0.03 to 0.07. In our analysis using an ordbeta regression with a logit link, the model predicts that in the absence of rain, the baseline probability of observing significant frugivory is approximately 17%. For each additional centimeter of monthly precipitation, the odds of observing greater frugivory increase by about 4.1%. The probability of direction (pd) for this association is 100%, indicating a certain positive association between lagged monthly precipitation and frugivory in the gopher tortoise (Makowski et al. 2019). The model's Rhat value of 1.00 confirms convergence and an effective sample size (ESS) of 16,160 suggests high reliability in this estimate.

### 3.3 Functional Shift in Seed Dispersal With Increasing Frugivory

Of the total 17,886 seeds ingested, we recorded a total of 13,619 intact seeds dispersed by the gopher tortoise. A total of 62 seed species were identified (Table 2), with at least one species per dispersal syndrome from Table 1. However, some dispersal syndromes had a greater frequency of occurrence in the fecal samples than others. Foliage is the fruit species that had a frequency of occurrence of 98.07%, followed by seeds with the autochory (76.81%) and endozoochory (50.72%) syndromes, respectively. Although seeds exhibiting the synzoochory syndrome were ingested, all seeds (*n* = 3) were visibly damaged in the digestive process and were thus excluded from further analyses.

**TABLE 2:** Summary table listing all seeds that were identified to the species level along with their taxonomic family, dispersal syndrome (using criteria from Table 1), total seeds dispersed across all fecal samples (*n* = 207), the percent of all seeds dispersed that the species represents, the number of samples they are present in, and their frequency of occurrence.

Seed species dispersed summary table					
Syndromes and species	Taxonomic family	Number of samples present in	Frequency of occurrence	Number of intact seeds dispersed	Percent of all intact seeds dispersed
Anemochory	—	4	1.93%	33	0.24%
*Andropogon glomeratus*	Poaceae	2	0.97%	2	0.01%
*Casuarina equesitifolia*	Casuarinaceae	1	0.48%	30	0.22%
*Schizachyrium gracile*	Poaceae	1	0.48%	1	0.01%
Autochory	—	159	76.81%	6267	46.13%
*Buchnera americana*	Orobanchaceae	1	0.48%	1000	7.36%
*Chamaecrista deeringiana*	Fabaceae	2	0.97%	1	0.01%
*Chamaecrista nictitans var aspera*	Fabaceae	2	0.97%	5	0.04%
*Euphorbia cyathophora*	Euphorbiaceae	8	3.86%	68	0.50%
*Euphorbia heterophylla*	Euphorbiaceae	1	0.48%	2	0.01%
*Euphorbia hirta*	Euphorbiaceae	60	28.99%	3373	24.83%
*Euphorbia hypericifolia*	Euphorbiaceae	29	14.01%	1407	10.36%
*Indigofera spicata*	Fabaceae	17	8.21%	65	0.48%
*Indigofera suffruticosa*	Fabaceae	1	0.48%	5	0.04%
*Leucaena leucocephala*	Fabaceae	1	0.48%	1	0.01%
*Malvastrum coromandelianum*	Malvaceae	6	2.90%	12	0.09%
*Melanthera parvifolia*	Asteraceae	4	1.93%	13	0.10%
*Piloblephis rigida*	Lamiaceae	1	0.48%	1	0.01%
*Richardia grandiflora*	Rubiaceae	19	9.18%	240	1.77%
*Richardia scabra*	Rubiaceae	1	0.48%	4	0.03%
*Vachellia farnesiana*	Fabaceae	1	0.48%	2	0.01%
*Waltheria indica*	Malvaceae	5	2.42%	68	0.50%
Endozoochory	—	105	50.72%	3709	27.30%
*Byrsonima lucida*	Malpighiaceae	7	3.38%	563	4.14%
*Cassytha filiformis*	Lauraceae	3	1.45%	3	0.02%
*Coccothrinax argentata*	Arecaceae	3	1.45%	41	0.30%
*Guettarda scabra*	Rubiaceae	3	1.45%	63	0.46%
*Lantana camara*	Verbenaceae	9	4.35%	60	0.44%
*Metopium toxiferum*	Anacardiaceae	1	0.48%	0	0.00%
*Miconia bicolor*	Melastomataceae	5	2.42%	1129	8.31%
*Momordica charantia*	Cucurbitaceae	3	1.45%	24	0.18%
*Morinda royoc*	Rubiaceae	1	0.48%	1	0.01%
*Mosiera longipes*	Myrtaceae	2	0.97%	1000	7.36%
*Opuntia austrina*	Cactaceae	12	5.80%	272	2.00%
*Physalis walterii*	Solanaceae	2	0.97%	10	0.07%
*Sabal palmetto*	Arecaceae	17	8.21%	48	0.35%
*Serenoa repens*	Arecaceae	35	16.91%	477	3.51%
*Vaccinium myrsinites*	Ericaceae	2	0.97%	18	0.13%
Epizoochory	—	35	16.91%	240	1.77%
*Desmodium incanum*	Fabaceae	8	3.86%	55	0.40%
*Desmodium triflorum*	Fabaceae	1	0.48%	6	0.04%
*Sida acuta*	Malvaceae	1	0.48%	7	0.05%
*Sida rhombifolia*	Malvaceae	8	3.86%	51	0.38%
*Sida ulmifolia*	Malvaceae	9	4.35%	49	0.36%
*Stylosanthes hamata*	Fabaceae	8	3.86%	72	0.53%
Foliage is the Fruit	—	203	98.07%	3037	22.35%
*Alysicarpus vaginalis*	Fabaceae	43	20.77%	791	5.82%
*Dactyloctenium aegyptium*	Poaceae	2	0.97%	17	0.13%
*Dichanthelium aciculare*	Poaceae	122	58.94%	2057	15.14%
*Digitaria ciliaris*	Poaceae	8	3.86%	50	0.37%
*Eustachys petrea*	Poaceae	1	0.48%	23	0.17%
*Panicum maximum*	Poaceae	2	0.97%	31	0.23%
*Paspalum caespitosum*	Poaceae	5	2.42%	6	0.04%
*Paspalum malacophyllum*	Poaceae	6	2.90%	28	0.21%
*Paspalum monostachyum*	Poaceae	1	0.48%	1	0.01%
*Paspalum notatum*	Poaceae	2	0.97%	5	0.04%
*Paspalum setaceum*	Poaceae	2	0.97%	1	0.01%
*Rhynchospora floridensis*	Cyperaceae	1	0.48%	1	0.01%
*Rhynchospora grayii*	Cyperaceae	2	0.97%	1	0.01%
*Spermacoce verticillata*	Rubiaceae	5	2.42%	18	0.13%
*Tripsacum floridanum*	Poaceae	1	0.48%	7	0.05%
Hydrochory	—	3	1.45%	89	0.66%
*Cyperus filiculmis*	Cyperaceae	3	1.45%	89	0.66%
Myrmecochory	—	14	6.76%	244	1.80%
*Croton glandulosus var septentrionalis*	Euphorbiaceae	1	0.48%	2	0.01%
*Croton linearis*	Euphorbiaceae	4	1.93%	23	0.17%
*Piriqueta cistoides subsp caroliniana*	Turneraceae	3	1.45%	195	1.44%
*Turnera ulmifolia*	Turneraceae	6	2.90%	24	0.18%
Synzoochory	—	3	1.45%	0	0.00%
*Quercus pumila*	Fagaceae	3	1.45%	0	0.00%

**FIGURE 4:**
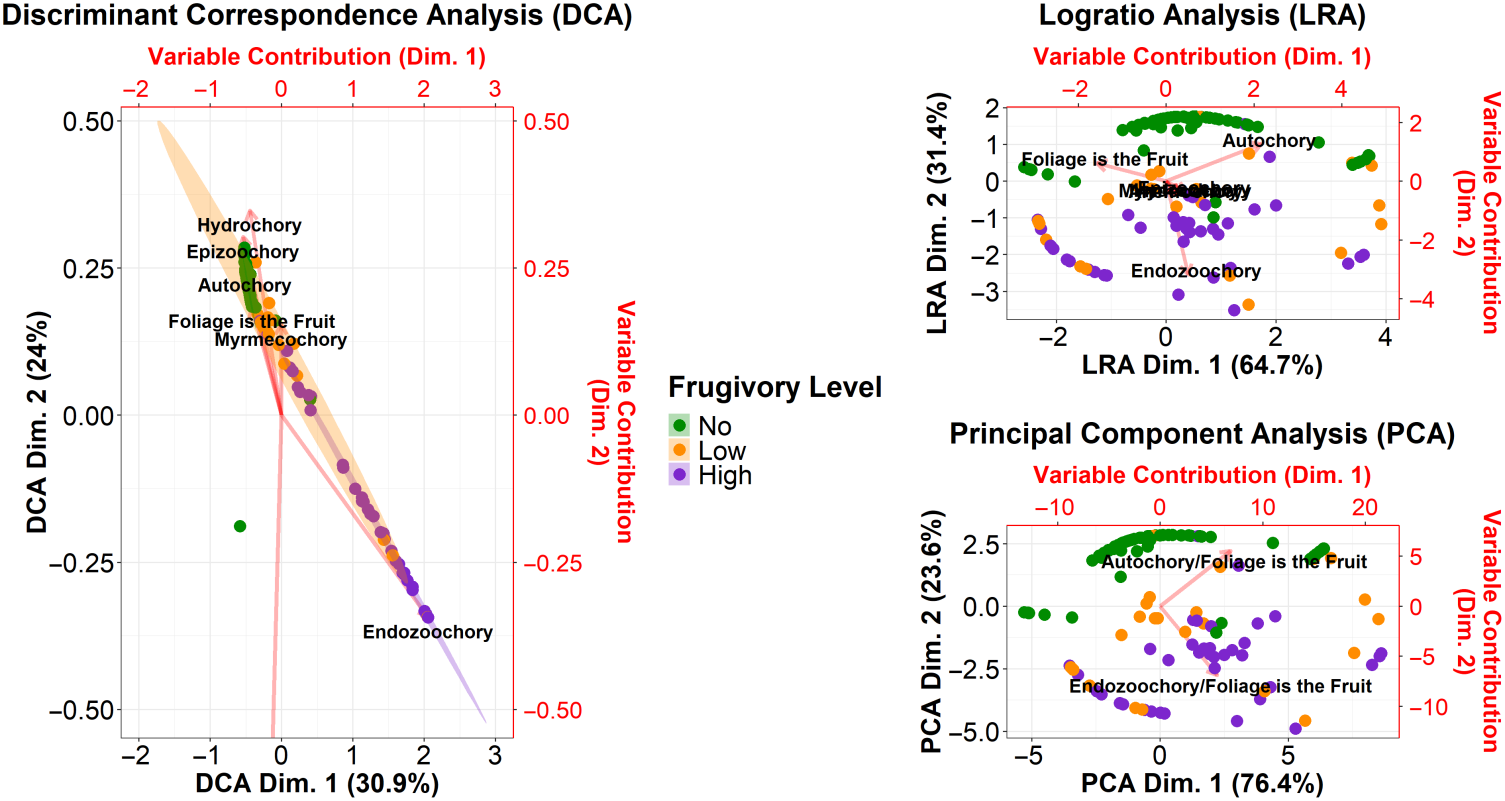
Correspondence analysis (CA) illustrating the influence of frugivory on the plant syndromes dispersed by the gopher tortoise. The 99% confidence intervals are drawn around the “No” (green), “Low” (orange), and “High” (purple) frugivory levels. The red arrows depict the eigenvectors for each dispersal syndrome, indicating how they change in relation to frugivory. The dispersal syndromes are labeled in the following fashion, “endo” = Endozoochory, “myrme” = Myrmecochory, “foliage” = Foliage is the Fruit, “anemo” = Anemochory, “epizoo” = Epizoochory, “auto” = Autochory, “hydro” = Hydrochory.

The CA was ultimately performed on 188 of the 207 samples because the remaining 19 samples either did not have seeds or only had visibly damaged seeds. The CA illustrated clear differences in the syndromes dispersed as frugivory increased (Figure 4). The 99% confidence ellipses in the CA provide a visual representation of variability and significant changes in the dispersal syndromes associated with frugivory levels. The No and Low frugivory level ellipses indicated increased dispersal of seeds with the myrmecochory and foliage is the fruit syndromes, while the No frugivory level ellipse was stretched in the direction of the autochory and hydrochory eigenvectors—indicating dispersal of those syndromes as well. When the tortoises exhibited low levels of frugivory, they dispersed a relatively even distribution of seed syndromes, whereas when they exhibited high levels of frugivory, they mainly dispersed seeds with the endozoochory syndrome. This observed shift in plant syndromes dispersed depicts seed dispersal behavior more aligned with that of a primarily frugivorous seed disperser than that of an herbivorous one, as the gopher tortoise species is.

The PERMANOVA returned a statistically significant result (*p*‐value = 9.99E‐05***), indicating significant differences in the plant syndromes dispersed between the three levels of frugivory (Table 3). The PERMISP and Tukey post hoc tests further confirmed that there was a significant difference in Low‐High comparison and an even stronger difference in the syndromes dispersed between the No‐High frugivory comparison (Table 3). These combined results suggest that the suite of plant syndromes dispersed indeed shifts during periods of greater frugivory.

**TABLE 3:** Summary table containing the results of the permutational analysis of variance (PERMANOVA) on the left and the multivariate homogeneity of group dispersions analysis (PERMDISP) on the right.

Multivariate analyses summary tables										
PERMANOVA summary table						PERMDISP summary table				
Parameters	Df	Sum of squares	*R*‐squared	*F*‐statistic	*p*	Frugivory level comparisons	Difference	Lower 95% Conf.	Upper 95% Conf.	Adjusted *p*
Frugivory level	2	8.551	0.136	14.565	9.99E‐05***	No‐High	0.083	0.018	0.148	0.008**
Residual	185	54.309	0.864			Low‐High	0.098	0.010	0.187	0.025*
Total	187	62.860	1			Low‐No	0.015	−0.062	0.092	0.885

*Note:* The PERMANOVA summary table contains the model parameters, degrees of freedom (Df), sum of squares, *R*‐squared, *F* statistic, and *p*‐value, while the PERMDISP table contains the frugivory level comparisons, difference between the groups, lower 95% confidence interval, upper 95% confidence interval, and adjusted *p*‐values. Signif. codes: 0 “***” 0.001 “**” 0.01 “*” 0.05 “.” 0.1 “1.”

## 4 Discussion

The findings of this study reveal that frugivory in the gopher tortoise is aligned with seasonal precipitation and strongly influences the dispersal of seeds exhibiting different syndromes. The 3‐month time lag between peak precipitation and frugivory likely reflects the time required for the production and availability of fleshy fruits following rainfall, consistent with previous studies indicating that frugivory in various species is temporally linked to the phenology of fruit‐bearing plants (Remis 1997; Herrera et al. 2008), which is often triggered by seasonal rainfall (Bancroft, Bowman, and Sawicki 2000; Redwine et al. 2007).

In the case of the western lowland gorilla, seasonal patterns of fleshy fruit availability shift its diet from a primarily folivorous to a more frugivorous one (Remis 1997). These seasonal dietary shifts toward frugivory alter the behavior and movement patterns of this species during periods of fleshy fruit abundance (Robira et al. 2023). Furthermore, frugivorous bats use seasonal increases in fleshy fruit availability to diverge their resource use patterns from conspecifics, thereby resulting in a greater incidence of individual diet specialization (Herrera et al. 2008)—a phenomenon recently confirmed to be occurring with the tortoises of this study (Figueroa et al. 2024), as seasonal fluctuations in rainfall subsequently trigger fleshy fruit production in the local plant communities (Bancroft, Bowman, and Sawicki 2000, Redwine et al. 2007).

The strong correlation between lagged precipitation and frugivory suggests that gopher tortoises track seasonal fleshy fruit availability, adjusting their diet accordingly to maximize fruit consumption when it is most abundant. This behavior may be exhibited as a result of increased ecological opportunity (Herrera et al. 2008; Araújo, Bolnick, and Layman 2011; Figueroa et al. 2024), to reduce intraspecific resource competition (Bolnick et al. 2003), or to meet energetic demands (Bury and Germano 1994). The seasonal tracking of fleshy fruits is not very surprising as it has been observed in other species (Remis 1997; Koike et al. 2008; Abrahms et al. 2021), but its implications for community‐wide seed dispersal are important to consider.

While dispersal syndromes alone may be unreliable for predicting animal‐mediated seed dispersal (Green, Baltzinger, and Lovas‐Kiss 2021), the adaptation of fleshy fruits may indeed confer competitive advantages by not only increasing seed dispersal for fleshy‐fruited species, but decreasing seed dispersal for competing species that exhibit other syndromes. Interestingly, the dispersal syndrome that was most strongly (negatively) affected by increased frugivory in this study was the foliage is the fruit syndrome (Janzen 1984). This syndrome is suggested to have evolved in many herbaceous plants to increase ingestion of their seeds by large herbivores. These plants offer a nutritious reward of foliage that is contaminated with seeds in order to coax herbivores into dispersing their seeds. This syndrome is prevalent in the Poaceae plant family and is found in species of Fabaceae (Janzen 1984). Considering that both plant families are important in the gopher tortoise diet (Birkhead et al. 2005; Ashton and Ashton 2008; Figueroa, Lange, and Whitfield 2021), the insights from this study could have broad implications for better understanding the seed dispersal ecology of seeds exhibiting this syndrome.

Although the gopher tortoise exhibits flexibility in its diet (Ashton and Ashton 2008), its effectiveness as a seed disperser for various plant syndromes is enhanced when it consumes more fleshy fruits. The significant results from the PERMANOVA and subsequent analyses underscore the distinct differences in the plant syndromes dispersed across varying levels of frugivory. Our study demonstrates that as the gopher tortoise becomes more frugivorous, its functional role as a seed disperser shifts substantially. At high levels of frugivory, the gopher tortoise not only primarily disperses seeds with the endozoochory syndrome, but also drastically reduces the seeds they disperse that exhibit the foliage is the fruit syndrome. These observations not only suggest a shift in foraging strategy, but a functional shift toward a role better characterized as that of a classical frugivore (Jordano 2000; Levey, Silva, and Galetti 2002), which can be thought of as a conceptual shift along the mutualism‐antagonism continuum illustrated in van Leeuwen et al. (2022).

The ability of gopher tortoises to adapt their diet in response to seasonal changes in fruit availability underscores their crucial role as seed dispersers for the critically imperiled pine rockland plant community of South Florida and the plant communities of other ecosystems they inhabit (Auffenberg and Franz 1982; Figueroa, Lange, and Whitfield 2021; Whitfield et al. 2022). By dispersing a wide range of seeds, including those from fleshy fruits and other syndromes, they contribute to maintaining plant diversity and ecosystem function (McConkey et al. 2012; Howe 2016). The identified lag in frugivory following precipitation highlights the tortoises' capacity to exploit temporal resource peaks, which is crucial for the regeneration of the pine rockland flora in periods where disturbances such as hurricanes and fires may result in open habitat for colonization (Snyder 1991; Henry et al. 2020).

Moreover, the predominance of fleshy fruit seed dispersal during periods of high frugivory suggests that gopher tortoises can significantly influence the recruitment and spatial distribution of fleshy fruit‐bearing plants. This functional shift may have broader implications for the dynamics of plant communities, potentially enhancing the competitive advantage of plants with the endozoochory syndrome over others with competing dispersal syndromes such as the foliage is the fruit syndrome. Gopher tortoises are already known to be effective seed dispersers for many plant species (Richardson and Stiling 2019a), with their use of movement corridors directing the dispersal of seeds to suitable sites for germination (Hanish 2018).

Although herbivory may mediate plant community succession (Heinen and Castillo 2019), and alter species richness and diversity via nonselective foraging (Richardson and Stiling 2019b; Ceballos and Goessling 2023), the gopher tortoise likely has an understated impact on plant communities via seed dispersal. The impacts of the gopher tortoise via seed dispersal can be gleaned from the diversity of seeds dispersed in this and previous studies (Carlson, Menges, and Marks 2003; Birkhead et al. 2005; Figueroa, Lange, and Whitfield 2021), as well as in the habitat associations of this species which include areas with open canopy and sandy soils (Whitfield et al. 2022)—often favorable conditions for seed germination.

For fleshy‐fruited species whose seeds have a high frequency of occurrence, the gopher tortoise may be a reliable seed disperser by consistently consuming their fruits and dispersing their seeds. However, even for species whose seeds may not have been dispersed as frequently, the gopher tortoise may still be an effective seed disperser by gorging on their fruits which may be more temporally restricted in availability but are consumed in exorbitant amounts, as in the case of the state‐threatened locustberry ( *Byrsonima lucida* ), West Indian lilac (*Miconia bicolor*), and longstalked‐stopper ( *Mosiera longipes* ), resulting in a narrow window of active seed dispersal. As a result, the conservation of the gopher tortoise is not only in service of protecting this chelonian, but it is in service of protecting the commensals that use their burrows (Diemer 1986; Lips 1991; Melanson 2021), and the thousands of plant species with which it interacts (Ashton and Ashton 2008).

While this study provides a comprehensive analysis of frugivory and seed dispersal in the gopher tortoise, several limitations should be acknowledged. The reliance on scat analysis, although effective, may not capture all aspects of the tortoises' diet or the fate of dispersed seeds. Future studies could quantify the availability of plants exhibiting different dispersal syndromes or integrate tracking of seed fates from dispersal to germination and establishment (Godoy and Jordano 2001; Schupp and Jordano 2011), providing a more holistic understanding of the ecological impacts of gopher tortoise as a seed disperser for various species. Additionally, a thorough quantification of seed dispersal effectiveness for the gopher tortoise could provide insight into how effective it is as a dispersal vector, opening the possibility for comparative studies along the mutualism‐antagonism continuum (*sensu* van Leeuwen et al. 2022). Finally, long‐term studies examining interannual variability in precipitation and frugivory would also be valuable in understanding the impacts of climate change on these dynamics.

## Author Contributions

**Adrian Figueroa:** conceptualization (lead), data curation (lead), formal analysis (lead), funding acquisition (equal), investigation (lead), methodology (lead), project administration (lead), resources (equal), supervision (lead), validation (equal), visualization (equal), writing – original draft (lead), writing – review and editing (lead). **Pavel Chernyavskiy:** formal analysis (equal), methodology (equal), supervision (lead), validation (lead), visualization (equal). **Michael Greenacre:** formal analysis (equal), methodology (equal), supervision (lead), validation (lead), visualization (equal). **Alyssa Herrera:** data curation (supporting), investigation (equal), methodology (equal), validation (equal). **Lydia Cuni:** investigation (equal), methodology (equal), resources (equal), validation (equal). **Jennifer Villate:** investigation (supporting), methodology (supporting). **Mauro Galetti:** conceptualization (equal), supervision (equal), validation (equal), visualization (equal). **Hong Liu:** conceptualization (equal), supervision (lead), visualization (equal), writing – original draft (supporting), writing – review and editing (supporting). **Steven Whitfield:** conceptualization (equal), funding acquisition (equal), investigation (equal), project administration (supporting), resources (lead), supervision (lead), writing – original draft (supporting), writing – review and editing (supporting).

## Conflicts of Interest

The authors declare no conflicts of interest.

## Supporting information


Data S1.

## Data Availability

The datasets used and/or analyzed during the current study are available from the corresponding author on reasonable request.
