# Supplementary material for: Precipitation Drives Frugivory in a Subtropical Generalist Herbivore, the Gopher Tortoise, and Alters Its Functional Role as a Seed Disperser
Source: Ecol Evol. 2024 Nov 18;14(11):e70585. doi: 10.1002/ece3.70585 (PMC11573422; doi:10.1002/ece3.70585)
Supplement: Supplementary file 1 — Data S1. [file ECE3-14-e70585-s001.docx]

**Manuscript Title:** Precipitation Drives Frugivory in a Subtropical Generalist Herbivore, the Gopher Tortoise, and Alters its Functional Role as a Seed Disperser

**Supplementary Materials**

**Seed Extrapolation Methods:**

To reliably extrapolate the number of small seeds in fecal samples, where seed counts were likely in the hundreds, we developed a resampling technique to train our visual estimation method. In this technique, we manually extracted all visible seeds from the fecal sample and placed them in a small petri dish with a field of view of 2.70 cm². The surface area of the petri dish was calculated to be 70.88 cm². We then divided the surface area of the petri dish by the field of view of the microscope to determine the number of independent observations that could be made (approximately 26).

We randomly shook the petri dish, ensuring no seeds fell out, and counted all visible seeds in the field of view of the microscope. This process was repeated 30 times to obtain a statistically sound estimate of the number of seeds in the sample. After completing this exercise, we calculated the mean and standard error of the seeds counted during the resampling technique.

Initially, we employed this technique as needed. However, as we became more familiar with the estimation process, we found it prudent to round the seed counts to the nearest hundred. This approach was subsequently adopted for further seed extractions when seed counts for small-seeded species were very high.
